# Exploiting of Green Synthesized Metal Oxide Nanoparticles for Spectrophotometric Determination of Levofloxacin, Cephalexin, and Cefotaxime Sodium in Commercial Products

**DOI:** 10.3390/nano11051099

**Published:** 2021-04-24

**Authors:** Nawal Ahmed Alarfaj, Wedad Altuhami Al-Onazi, Amal Mohammed Al-Mohaimeed, Maha Farouk El-Tohamy, Hadeel Abdulaziz Alabdulmonem

**Affiliations:** 1 Department of Chemistry, College of Science, King Saud University, P. O. Box 22452, Riyadh 11495, Saudi Arabia; nalarfaj@ksu.edu.sa (N.A.A.); walonazi@ksu.edu.sa (W.A.A.-O.); muhemeed@ksu.edu.sa (A.M.A.-M.); h-chemistry@hotmail.com (H.A.A.); 2 Department of Chemistry, College of Science, Princess Nourah Bint Abdulrahman University, P. O. Box 84428, Riyadh 11671, Saudi Arabia

**Keywords:** levofloxacin, cephalexin, cefotaxime sodium, metal oxide nanoparticles, green chemistry

## Abstract

In this study, two metal oxide nanoparticles NiO and MnO_2_ were synthesized from green sources *Mentha spicata* (*M. spicata*) extract and *Malus domestica* (*M. domestica)* peel extract, respectively. The optical and physical properties of the synthesized nanoparticles were characterized using spectroscopic and microscopic techniques. Simple, precise, and new spectrophotometric probes were suggested for the determination of three cephalosporin antibiotics, including levofloxacin (LVX), cephalexin (CPX), and cefotaxime sodium (CTX) in their pure form and commercial products. The spectrophotometric detection of the selected drugs is based on the catalytic enhancement of NiO and MnO_2_ nanoparticles (NPs) due to their unique optical properties. Linear relationships with main correlation coefficients 0.999 were obtained at 0.1–20, 1.0–80, and 0.001–100 µg mL^−1^ for the three drugs in the presence of NiONPs, whereas 0.01–60, 0.1–160, and 0.01–80 µg mL^−1^ were obtained in the presence of MnO_2_NPs at absorption wavelengths 290, 262, and 235 nm for LVX, CPX and CTX, respectively. The analytical methods were validated and successfully used for determination of the instigated drugs in their bulk and commercial dosage forms.

## 1. Introduction

Infectious diseases are one of the major human disorders that caused by various microorganisms such as bacteria, viruses, fungi, or parasites. The infection can be transferred directly or indirectly from patient to another or due to the exposure to other infected animals. The failure of treatment of the infectious diseases with medications by using unsuitable drugs or uncontrolled dose is a leading cause of death especially in young children worldwide. Untreated infectious diseases are still a serious social and medical challenge, particularly in many developing countries [1]. 

Nowadays, various antibiotics or antibacterial drugs are recommended to destroy or slow down the growth of Gram-positive and Gram-negative microorganisms [2]. Among these drugs are fluoroquinolones and cephalosporins. Fluoroquinolones are broad-spectrum bactericidal medications often used for genitourinary, hospital-acquired, and community-acquired infections. They are recommended as first-line therapy [3]. However, cephalosporins are a large bactericidal group of antibiotics derived from the mold Acremonium, which act by the similar pathway of penicillins [4]. 

The current study concerns with three different antibiotics belonging to fluoroquinolones (Levofloxacin, LVX) and cephalosporins (Cephalexin monohydrate, CPX and Cefotaxim sodium, CTX). LVX and CPX are oral active drugs used in the treatment of a variety of bacterial infections. LVX acts by inhibiting the enzymes responsible for DNA replication; however, CPX acts by inhibiting the cross linkage between *N*-acetyl muramic acid and *N*-acetylglucosamine in the bacterial cell wall causing cell lysis. Furthermore, CTX is a parenteral active cephalosporin antibiotic that acts by inhibiting the bacterial cell wall synthesis [5]. 

Several analytical methods have been reported for the determination of the investigated drugs, including spectrophotometric methods [6,7,8], spectrofluorometric methods [9,10,11], electrochemical detection such as voltammetric methods [12,13,14], and various chromatographic separation methods [15,16,17]. 

Although separation techniques provide a fast, automated, and accurate determination of chemical substances, they still possess many drawbacks, such as the consumption of large amounts of solvents and reagents, which are costly and require high analyst skills. Moreover, electrochemical techniques can also be carried out quickly; the outcomes are recorded as electrical signals, but unfortunately, these approaches exhibited some errors in analysis, necessitating environmental protection to lower the toxicity. However, the spectroscopic techniques, particularly spectrophotometry as a quantitative measurement technique still gained much opportunity and attention, due to its simplicity, high throughput, stability, and sensitivity [18,19]. 

Nanotechnology becomes one of the most prominent areas of scientific research in material science and biotechnology. Many researchers started to concentrate their vision in investigating the unique characteristics of nanomaterials and their tremendous applications [20]. With progress in all areas of industry and technology, the interest has been focused on nanoscale materials, which possess new physical and chemical properties due to the change in their size and morphology [21]. 

Nanomaterials are widely used in various life fields including therapeutic and biomedical applications. The main advantages of nanoparticles are increasing aqueous solubility by improving the bioavailability, increasing resistance time in the body by elevating the half-life of receptors clearance and enhance the drug targeting to specific location in the body. In addition, they reduce the required quantity of toxic drugs and enable the safe delivery of toxic therapeutic drugs with the protection of nonspecific tissues and cells from potential diverse effects [22]. 

Within the functional materials that can be synthesized in the nanometric scale are metal oxides, which have distinctive properties such as high electrical conductivity, excellent mechanical stability, better chemical reactivity, and enhanced catalytic activity [23]. 

Recently, nickel oxide (NiO) and manganese dioxide (MnO_2_) nanomaterials have gained great attention due to their excellent physical and chemical features. NiO is an important p-type semiconductor transition metal oxide with cubic lattice structure and large spin optical density. It has been extensively used in various applications such as electrochemical sensors [24], electronic films [25], catalysis [26], batteries [27], and biomedical applications [28,29]. In addition, due to the extraordinary properties of MnO_2_NPs, they have been used in different applications including, batteries, catalysis, magnetic materials, electronic devices, and pharmaceutical analysis [30,31,32,33,34]. Several methods have been attempted to synthesize NiO and MnO_2_ nanoparticles, including wet chemical synthesis [35,36], the sol–gel method [37,38], and thermal decomposition [39,40]. Interestingly, the morphology of the engineered metal oxides, e.g., shape and size, can be modulated by varying the conditions, chemicals, and concentrations used. However, the obtained nanoparticles can suffer from certain limitations such as lack of understanding the mechanism of modulation, instability, bioaccumulation, toxicity, and the need for high skill operators [41]. Therefore, the aforementioned limitations opened new and great opportunities in the research field to explore the use of green chemistry to improve the economics of chemical manufacturing and to enhance the environmental protection. The green chemistry concept presents an attractive technology to chemists, researchers, and industrialists for innovative chemistry research and applications. It is characterized as a reduction of the environmental damage accompanied by the production of materials and respective minimization and proper disposal of wastes generated during different chemical processes [42]. The use of plant extracts in the biosynthesis of metal and metal oxide nanoparticles involves the presence of secondary metabolites as reducing agents. Commonly, biomolecules in plant extracts act as reducers, stabilizers, or both in the process of forming nanoparticles. Moreover, the same plant extract can be used to synthesize various types of nanoparticles; e.g., citrus lemon has been used for the green synthesis of silver nanoparticles and ZnO nanoparticles [43,44].

The literature survey addressed many reports concerned with the green synthesis of nanoparticles [45,46] and their prospective in various applications such as electrochemical sensors [47,48], photocatalysis [49,50] and pharmaceutical analysis [51,52]. 

This study described the use of natural sources such as *Mentha spicata* (*M. spicata*) extract and *Malus domestica* (*M. domestica)* peel extract for the synthesis of NiONPs and MnO_2_NPs, respectively. The synthesized nanoparticles were fully characterized using various spectroscopic and microscopic techniques. In addition, the prepared metal oxide nanoparticles were employed for the spectroscopic quantification of three different antibiotics, LVX, CPX, and CTX, in their bulk powders and commercial dosage forms. 

## 2. Materials and Methods

### 2.1. Chemicals

All chemicals are pure grade and used without further purification. Sigma Aldrich (Hamburg, Germany) supplied nickel nitrate hexahydrate (99.9%), potassium permanganate (99.0%), sodium hydroxide (98.0%), methanol (99.9%), ethanol (99.9%), and acetonitrile (99.9%). Pure grade of each of LVX, CPX, and CTX was kindly supplied by Tabuk pharmaceutical Co., Tabuk, Saudi Arabia. The pharmaceutical preparations, including Tavanic^®^ 500 mg LVX/tablet (SanofiWinthrop Industries, Le Trait, France), Keflex^®^ 500 mg CPX/tablet (Facta Farmaceutical, Milano, Italy), and Foxim^®^ 1 g CTX/5 mL vial (Tabuk pharmaceutical Co., Tabuk, Saudi Arabia) were purchased from local drug stores (Riyadh, Saudi Arabia).

### 2.2. Instruments

All spectrophotometric measurements and the characterization of the synthesized nanoparticles were carried out by measuring the UV-Vis spectra of the as-prepared nanoparticles using an Ultrospec 2100-Biochrom spectrophotometer, (Biochrom Ltd., Cambium, Cambridge, UK). However, Fourier transform infrared (FTIR) spectra of the formed nanoparticles were recorded using a PerkinElmer FTIR spectrophotometer (PerkinElmer Ltd., Yokohama, Japan). The morphology of NiONPs and MnO_2_NPs were measured by scanning electron microscope (JEM-2100F, JEOL Ltd., Tokyo, Japan) and JEM-1400 transmission electron microscope (JEOL Ltd., Akishima, Tokyo, Japan). Energy-dispersive X-ray (EDX, JSM-7610F; JEOL, Tokyo, Japan) at 20 kV was used to determine the elemental composition of NiONPs and MnO_2_NPs. Additionally, the XRD patterns of NiONPs and MnO_2_NPs were obtained by Siemens D-5000 diffractometer (Siemens, Erfurt, Germany). To control the pH conditions of the tested solutions, pH-meter Metrohm model 744 (Metrohm Co., Herisau, Switzerland) was used. Distilled water (GFL, Burgwedel, Germany) was used throughout the experimental study.

### 2.3. Preparation of Analytical Solutions 

#### 2.3.1. Preparation of Standard Solutions of LVX, CPX and CTX 

Stock solutions (1000 µg mL^−1^) of each pure LVX, CPX, and CTX were prepared separately by dissolving 0.1 g in 100 mL of distilled water. Serial dilutions were performed to prepare the working solutions in the concentration ranges of 0.1–20, 1.0–80 and 0.001–100 µg mL^−1^ for the analysis of LVX, CPX and CTX in the presence of NiONPs and 0.01–60, 0.1–160, and 0.01–80 µg mL^−1^ for the three drugs in the presence of MnO_2_NPs, respectively.

#### 2.3.2. Preparation of Tablet and Injection Samples

Not less than ten tablets of Tavanic^®^ 500 mg LVX/tablet and Keflex^®^ 500 mg CPX/tablet were weighed and finely powdered. An accurate amount of the powder equivalent to 0.1 g was transferred into a 100-mL conical flask. The contents were diluted to the mark with distilled water and sonicated for 10 min and then filtered using Whatman filter paper No. 41. The obtained solution was further diluted to the required concentrations using distilled water and then tested as mentioned under a general procedure. For Foxime^®^, 1 g of CTX/5 mL vial an equivalent volume to 1000 µg mL^−1^ was transferred to 100-mL volumetric flask and diluted to the mark with distilled water. The nominal content of each drug was determined using the calibration graph or the regression equation.

### 2.4. Preparation of *M. spicata* and *M. domestica* Extracts

*M. spicata* leaves and *M. domestica* peels were perfectly rinsed with distilled water and oven dried at 70 °C. The obtained biomass was ground into fine powder and kept at ambient temperature. The *M. spicata* extract was prepared by mixing 10 g of dried *M. spicata* biomass in 100 mL of distilled water boiled under constant stirring for 10 min. The content was cooled and filtered using Whatman No. 1 with pore size 25 µm filter paper. The resulted extract was stored in a refrigerator at 4 °C for further uses. 

The *M. domestica* peel extract was prepared by mixing 10 g of *M. domestica* peels biomass in 100 mL of distilled water and refluxed for 1 h at 70 °C. Cotton fabric was used to remove the solid particles from the formed suspension. The suspension was centrifuged for 10 min at 10,000 rpm and filtered using 0.2 µm filter (Millipore, Bedford, TX, USA), and the pH of the extract ranged between 3.7 and 4. The resulted extract was stored in a refrigerator at 4 °C for further use. Since the obtained aqueous extracts have various secondary metabolites containing hydroxyl and carbonyl groups, these functional groups allowed the plant extracts to serve as reducing and stabilizing agents for the green synthesis of NiONPs and MnO_2_NPs, respectively [53].

### 2.5. Green Synthesis of NiONPs and MnO_2_NPs

The NiO and MnO_2_ nanoparticles were synthesized using *M. spicata* and *M. domestica* peels extract, respectively. The synthesis of NiONPs was carried out under constant stirring by reducing 100 mL of nickel nitrate hexahydrate (0.1 mol L^−1^) using 50 mL of *M. spicat* extract as a reducing agent. Subsequently, 5 mL of sodium hydroxide solution (0.1 mol L^−1^) was added dropwise. The mixture was kept under magnetic stirring for 4 h at room temperature until the formation of NiONPs. The obtained nanoparticles were filtered using Whatman filter paper No. 41 and then washed with distilled water three times to remove any excess of sodium hydroxide. The obtained NiONPs were dried at 100 °C for 12 h and calcined at 350 °C for 3 h.

MnO_2_ nanoparticles were synthesized by adding 50 mL of (*M. domestica*) peels extract to 100 mL of potassium permanganate solution (0.1 mol L^−1^). Approximately 5 mL of 1.0 mol L^−1^ sodium hydroxide was added dropwise until the pH elevated to 8. The formed dark brown precipitate was filtered using Whatman filter paper No. 41, washed with distilled water, and calcined at 700 °C for 3 h.

The NiONPs and MnO_2_NPs stock solutions were prepared by adding 0.1 g of each nanopowder to 100 mL distilled water. The suspensions were sonicated for 10 min and stored in refrigerator at 4 °C. The suitable volumes of added nanoparticles were selected for further investigations by testing different volumes of the previously prepared nanoparticles (0.1 g/100 mL) in the ranges of 0.1–1.4 mL and 0.5–3.0 mL of NiONPs and MnO_2_NPs, respectively. It was found that the highest absorption maxima were achieved by adding 0.2, 0.6, and 0.4 mL of NiONPs and 2.0, 1.0, and 1.5 mL of MnO_2_NPs. Thus, these volumes were selected for further experimental analysis. 

### 2.6. General Analytical Procedure

#### General Analytical Procedure Using NiONPs and MnO_2_NPs

Aliquots of solutions with different concentrations of LVX (0.001–60 µg mL^−1^), CPX (1–80 µg mL^−1^), and CTX (0.001–100 µg mL^−1^) were transferred into a series of 10 mL volumetric flasks. Then, 0.2, 0.6, and 0.4 mL of NiONPs was added to LVX, CPX, and CTX, respectively. However, aliquot solutions of LVX (0.01–100 µg mL^−1^), CPX (0.01–160 µg mL^−1^), and CTX (0. 01–120 µg mL^−1^) were transferred into a series of 10 mL volumetric flasks and 2.0, 1.0, and 1.5 mL of MnO_2_NPs were added to LVX, CPX, and CTX, respectively. The absorbance of the solutions was measured at 290, 262, and 235 nm, respectively. 

## 3. Results

### 3.1. Characterization of NiONPs and MnO_2_NPs

**Figure 1:**
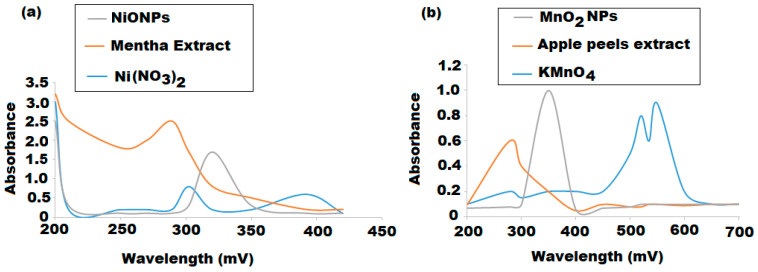
UV-Vis absorption spectra of synthesized (**a**) NiONPs and (**b**) MnO_2_NPs using *Mentha spicata* leaves and *Malus domestica* peel extracts, respectively.

Various spectroscopic and microscopic techniques were used to characterize the formation of NiONPs and MnO_2_NPs. The optical features of each synthesized metal oxide nanoparticles were studied using UV-Vis spectroscopy. The absorption spectra in Figure 1a showed two broad absorption peaks at 302 and 392 nm for the Ni(NO_3_)_2_ precursor and one absorption peak at 289 nm for *M. spicata* leaves extract. However, NiONPs displayed a significant absorption peak at 320 nm due to the unique property of surface plasmon resonance (SPR). The obtained results were in agreement with the literature [54]. In Figure 1b, the absorption spectra displayed a significant absorption peak at 550 nm related to potassium permanganate precursor, while another peak can be observed at 280 nm, which is corresponding to *M. domestica* extract, and the appeared peak at 350 nm was assigned to be corresponding to MnO_2_NPs. The obtained results matched those in the literature [55].

The band gap energy of the as-prepared NiONPs and MnO_2_NPs was calculated from the formula E_g_ = h*ν* where, E_g_ is the band gap energy, h is a blank constant, and ν is the frequency. The band gaps of NiONPs and MnO_2_NPs were calculated as 3.56 and 3.62 eV, respectively. The red shift of absorption spectrum to 320 and 350 nm for NiONPs and MnO_2_NPs, respectively and the decrease of band gap values revealed the formation of nanoscale materials. According to the literature [54,55], these distinct peaks represent the formation of NiONPs and MnO_2_NPs with particle sizes of approximately 50–100 nm, respectively. Additionally, in nanoscale materials, the relation between the band gap and the size diameter of particle is inversely proportional to each other. The higher the nanoparticles in size diameter, the lower in band gap value, due to quantization effect, but it never reaches zero [56]. 

**Figure 2:**
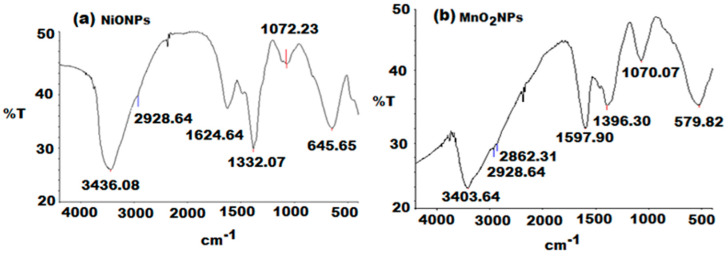
FTIR spectra in the range of 4000–500 cm^−1^ of green synthesized (**a**) NiONPs and (**b**) MnO_2_NPs using *Mentha spicata* and *Malus domestica* peel extract, respectively.

To ascertain the purity and nature of NiONPs surface, FTIR spectrum was recorded. As shown in Figure 2a, the broad absorption band at 645.65 cm^−1^ is assigned to Ni–O stretching vibration mode, and the broadness of the absorption band indicates that the NiO powder is a nanocrystal [57]. The presence of a broad band at 3436.08 cm^−1^ was attributed to O–H stretching vibration [58]. The weak band near 1624.64 cm^−1^ is assigned to H–O–H bending vibration mode, due to the adsorption of atmospheric moisture in air [59].

Another vibrational band that was observed at 1332.07 and 1072.23 cm^−1^ may be attributed to the carbonyl group C=O stretching vibration band. The serrated absorption bands in the region 1000–1500 cm^−1^ are assigned to the O–C=O symmetric and asymmetric stretching vibrations and the C–O stretching vibration [60], which indicated that the ultrafine powders tend to have strong physical absorption to H_2_O and CO_2_. The result of this FTIR spectroscopic study confirmed the reduction of nickel nitrate into NiONPs by using mint extract as reducing agent in the presence of NaOH. FTIR spectroscopy was also used to study the chemical composition of the MnO_2_NPs surface (Figure 2b).

The absorption band located at 579.82 cm^−1^ was ascribed to the typical collision O–Mn–O vibrations of the prepared MnO_2_ [61]. The recorded spectra revealed a broad band at 3403.64 cm^−1^ for O–H symmetric stretching frequency vibration and two bands at 2928.64 and 2862.31 cm^−1^ for C-H vibration stretching vibration bands [62]. Additionally, the bands at 1597.40 and 1396.30 cm^−1^ were corresponding to the existence of aromatic unsaturated C=C. Furthermore, the spectrum showed an absorption band at 1070.07 cm^−1^ due to the presence of C–O stretching vibration [62]. 

**Figure 3:**
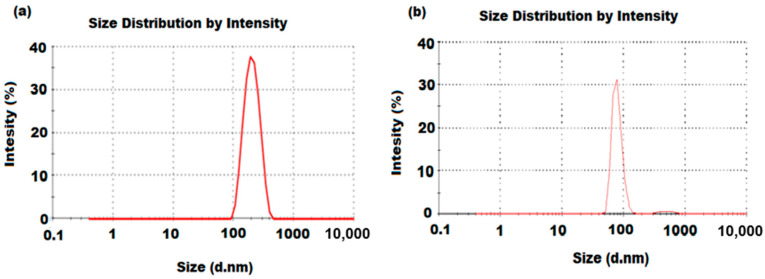
Particle size distribution of (**a**) NiONPs and (**b**) MnO_2_NPs synthesized by *Mentha spicata* and *Malus domestica* peel extract, respectively.

The particle size distributions of NiONPs and MnO_2_NPs were determined by a particle size analyzer (PSA). The obtained results showed that the particle size distributions were around 100 nm for both NiONPs and MnO_2_NPs, respectively (Figure 3a,b). The obtained results confirmed that the synthesized NiO and MnO_2_ using *M. spicata* leaf and *M. domestica* peel extracts are in nanoparticles form.

**Figure 4:**
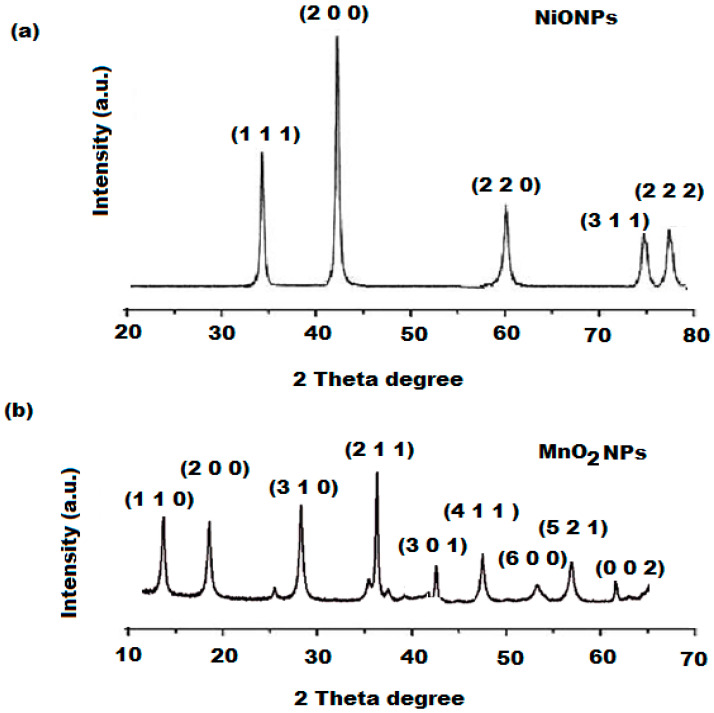
XRD spectra of the green synthesized (**a**) NiONPs and (**b**) MnO_2_NPs using *Mentha spicata* and *Malus domestica* peel extract, respectively.

X-ray diffraction patterns of NiONPs and MnO_2_NPs were recorded over a 2θ range of 20–80° to investigate the crystal structure of the as-prepared metal oxide nanoparticles. The phase purity of the NiO nanoparticles sample was studied using XRD analysis. The XRD pattern (Figure 4a) showed five different peaks at (2θ) of 34.17°, 42.26°, 62.73°, 75.29°, and 79.16° corresponding to (1 1 1), (2 0 0), (2 2 0), (3 1 1), and (2 2 2) planes, respectively. All these XRD peaks can be excellently indexed to the face-centered cubic crystalline shape of NiO, which are matched with that of the standard spectrum (JCPDS card number 04-0835) [63]. Debye–Scherrer formula was followed to calculate the grain size of the crystallites of NiONPs.

D = 0.9λ/βCosθ

where D is the crystal size, λ = 0.15418 nm is the wavelength used in the present study, and β is the Bragg angle θ of the X-ray diffraction peak. The average crystallite size of the as-synthesized nanoparticles was 88.63 nm. 

The XRD pattern of MnO_2_ nanoparticles (Figure 4b) displayed diffraction peaks at 2θ degree angles of 13.8°, 17.92°, 29.52°, 38.12°, 43.70°, 50.69°, 54.91°, 56.54°, and 62.15° corresponding to (1 0 0), (2 0 0), (3 1 0), (2 1 1), (3 0 1), (4 1 1), (6 0 0), (5 2 1), and (0 0 2) tetragonal structure. The recorded peaks are in good agreement with the JCPDS Card number of 44-0141 [64]. Debye–Scherrer formula was followed to calculate the particle size of the crystallites of MnO_2_NPs. The average crystallite size of the as-synthesized nanoparticles was 85.66 nm.

**Figure 5:**
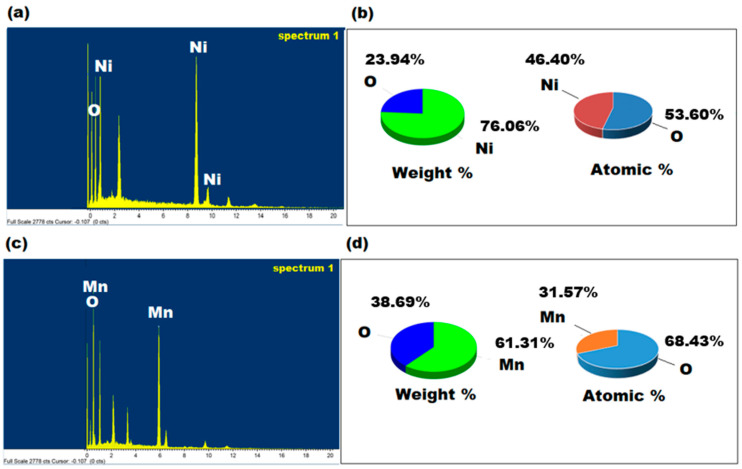
(**a**,**c**) EDX spectra of the green synthesized NiONPs and MnO_2_NPs using *Mentha spicata* and *Malus domestica* peel extracts, (**b**,**d**) represent the weight percentage and atomic percentage of the synthesized nanoparticles, respectively.

EDX analysis was used to quantify the chemical compositions of the green synthesized NiONPs and MnO_2_NPs using *M. spicata* and *M. domestica* extracts, respectively (Figure 5a,c). The results indicated that in NiONPs, the weight percentage of Ni and O were 76.06% and 23.94%, respectively. The atomic percentage was found to be Ni (46.40%) and O (53.60%). However, in case of MnO_2_NP, the weight percentage of Mn and O are 61.31% and 38.69%, respectively. The atomic percentage was found to be Mn (31.57%) and O (68.43%). No additional peaks corresponding to other elements were observed, revealing the purity of the synthesized NiONPs and MnO_2_NPs (Figure 5b,d).

**Figure 6:**
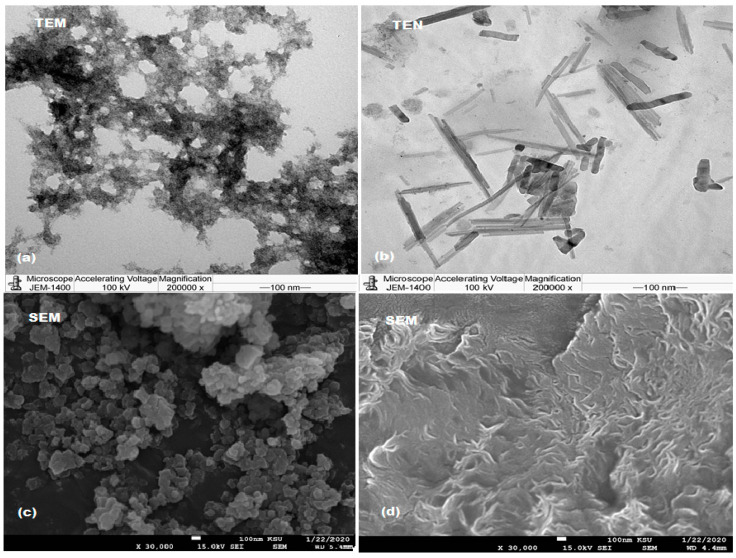
TEM (**a**,**b**) and SEM (**c**,**d**) images of green synthesized NiONPs and MnO_2_NPs using *Mentha spicata* and *Malus domestica* peel extracts, respectively.

The TEM images of the NiONPs (Figure 6a) indicated that the prepared metal oxide nanoparticles are fairly uniformly distributed, spherical in shape, and their sizes are between 50 and 100 nm. TEM images of MnO_2_NPs synthesized with the *M. domestica* extract showed the presence of short and long rods that are dense and agglomerated with particle size around 80–100 nm (Figure 6b).

Additionally, the surface morphology of NiONPs and MnO_2_NPs was investigated under SEM using 30,000× magnification. The image in (Figure 6c) showed the morphological structure of NiONPs, which appeared to be spherical and aggregated particles with particles size around 100 nm. The image in (Figure 6d) indicated that the MnO_2_NPs are nanorodes in the range 80–100 nm with significant aggregation that appeared to have a caddice clew morphology [65,66]. 

### 3.2. Optimum Experimental Conditions

Various experimental parameters were optimized, including the influence of the solvents, volume of nanoparticles, response time, and temperature. The conditions were optimized by varying one variable and observing its effect on the absorbance maxims of samples.

#### 3.2.1. Effect of Solvents on the Absorption Spectra

**Figure 7:**
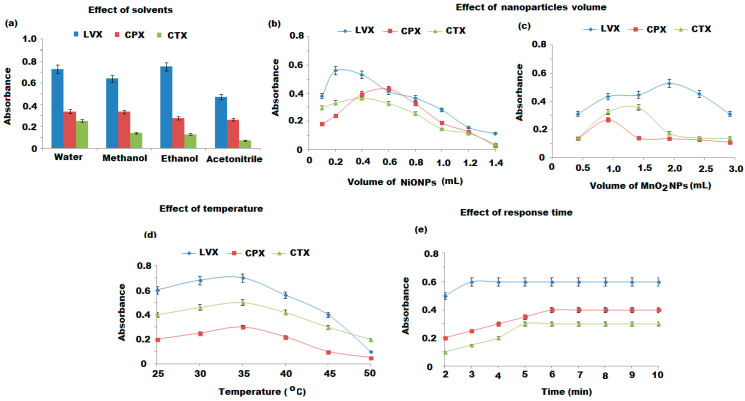
(**a**) Effect of solvents on the absorbance maxima of the three investigated drugs dissolved in different solvents and in the absence of nanoparticles, (**b**,**c**) effect of NiONPs and MnO_2_NPs volume, (**d**,**e**) effect of temperature 25–50 °C and effect of response time 2–10 min on the absorption of 10 μg mL^−1^ of LVX, CPX, and CTX solutions in the presence of the corresponding nanoparticles as indicated previously.

The UV-Vis absorption spectra of 10 µg mL^−1^ of each LVX, CPX, and CTX solution in the presence of different solvents such as water, methanol, ethanol, and acetonitrile were studied. The absorption maxima were recorded according to the solvent nature. Figure 7a showed the absorbance obtained at 290, 262, and 235 nm, respectively, of the three investigated drugs dissolved in different solvents, in the absence of nanoparticles.

#### 3.2.2. Volume of NiONPs and MnO_2_NPs

The effect of the volume of the as-prepared NiONPs and MnO_2_NPs in the ranges of (0.1–1.4 mL) and (0.5–3.0 mL) was investigated using 10 µg mL^−1^ of each LVX, CPX, and CTX solutions. The maximum absorption peaks were recorded after adding 0.2, 0.6, and 0.4 mL of NiONPs to LVX, CPX, and CTX, respectively, or by adding 2.0, 1.0, and 1.5 mL of MnO_2_NPs to the above-mentioned drugs, respectively. Thus, these volumes were used for further experimental studies (Figure 7b,c).

#### 3.2.3. Effect of Temperature and Response Time

The effect of temperature on the spectrophotometric determination of LVX, CPX, and CTX was studied by elevating the temperature of the tested samples and blank from 25 to 50 °C using water baths. It was observed that the absorbance of the three drugs was decreased by increasing the temperature more than 25–35 °C. Therefore, the suggested spectrophotometric method for the determination of the investigated drugs was performed at ambient temperature (Figure 7d). Further study was performed at various time intervals 2–10 min to evaluate the effect of response time between each drug sample and the added nanoparticles. The absorbance of each drug was recorded at 290, 262, and 235 nm for LVX, CPX, and CTX, respectively. Fast responses were noticed within 3, 6, and 5 min for the tested drugs in the presence of NiONPs and MnO_2_NPs, respectively. The optimized conditions for the suggested spectrophotometric method were presented in Table 1. 

### 3.3. Absorption Spectra

**Figure 8:**
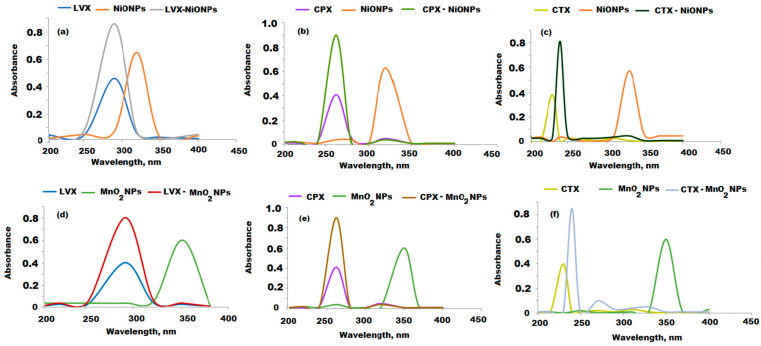
(**a**–**c**) The absorption spectra of 10 µg mL^−1^ of LVX, CPX, and CTX solutions in the presence of NiONPS and (**d**–**f**) in the presence of MnO_2_NPs, respectively.

The proposed spectrophotometric method based on the optical properties of the green synthesized metal oxide nanoparticles (NiONPs and MnO_2_NPs) was applied to determine LVX, CPX, and CTX. After 3, 6, and 5 min response time for 10 µg mL^−1^ of each drug with 0.2, 0.6, and 0.4 mL of NiONPs and 2.0, 1.0, and 1.5 mL of MnO_2_NPs, the absorbance was recorded at wavelengths 290, 262, and 235 nm, respectively. As shown in Figure 8a–f, the absorption spectra were significantly increased by 2-fold compared to the native spectra of the selected drugs. The spectrophotometric analysis could be suitable for the quantification of the tested drugs. 

### 3.4. Method Validation

The developed methods for the determination of the studied drugs were validated, and the data were reported with respect to ICH guidelines [67]. 

The linear relationships of the investigated drugs in the presence of NiONPs and MnO_2_NPs were determined by plotting the absorption vs. LVX, CPX, and CTX concentrations. The investigated drugs displayed linearity over the concentration ranges 0.1–20, 1.0–80, and 0.001–100 µg mL^−1^ for LVX, CPX, and CTX in the presence of NiONPs, whereas 0.01–60, 0.1–160, and 0.01–80 µg mL^−1^ were obtained in the presence of MnO_2_NPs (Table 2). The least square regression equations were derived as Y = 0.0492C + 0.0334 (r = 0.9986), Y = 0.0319C + 0.0699 (r = 0.9999), and Y = 0.0304C + 0.0312 (r = 0.9995) for LVX, CPX, and CTX in the presence of NiONPs and Y = 0.0464C + 0.0956, (r = 0.9974) Y = 0.0163C + 0.0489 (r = 0.9995), and Y = 0.0321C + 0.0835 (r = 0.9998) for the above-mentioned drugs in the presence of MnO_2_NPs.

The lower limits of detection and quantification (LOD) and (LOQ) of the investigated drugs were calculated by the equations at 3.3 *σ*/*s* and 10 *σ*/*s*, respectively, where σ represents the standard deviation of the response and s is the slope of the calibration graph. The recorded results showed that the LOD values were found to be 0.07, 0.09, and 0.0005 µg mL^−1^ for LVX, CPX, and CTX in the presence of NiONPs and 0.002, 0.006, and 0.0007 µg mL^−1^ for the three mentioned drugs in the presence of MnO_2_NPs. However, the obtained LOQ was found to be 0.21, 0.27, and 0.015 µg mL^−1^ for LVX, CPX, and CTX in the presence of NiONPs, while in the presence of MnO_2_NPs, LOQ were 0.006, 0.018, and 0.0021 µg mL^−1^ for the same drugs, respectively. 

The accuracy of the proposed spectrophotometric method was expressed as mean recoveries percentage ± standard deviation (mean ± SD) calculated from the obtained results. The developed methods showed good accuracy with percentage recoveries in the ranges of 99.27 ± 1.4, 99.59 ± 0.4, and 99.07 ± 0.8 for LVX, CPX, and CTX in the presence of NiONPs and 99.77 ± 0.5, 99.32 ± 0.7, and 99.17 ± 0.7 for the above-mentioned drugs, respectively (Table 3). 

In order to estimate the precision of the proposed method, intra-day and inter-day assays were applied. Three different concentrations of each drug were investigated in triplicate (*n* = 3), and the recorded data were calculated as relative standard deviation percentage (RSD %). As summarized in (Table 3), the calculated mean RSD % was found to be 1.5%, 0.7%, 0.6%, and 0.5%, 0.8%, and 0.7% for intra-day assay of LVX, CPX, and CTX in the presence of NiONPs and MnO_2_NPs, respectively. However, the inter-day assay displayed mean RSD % of 0.2%, 1.5%, 0.9% and 1.2%, 1.2%, and 0.9% of the above tested drugs in the presence of NiONPs and MnO_2_NPs, respectively. These results are less than 2%, revealing the high precision of the developed method.

Under optimized experimental conditions, the selectivity of the developed spectrophotometric method for the determination of LVX, CPX, and CTX in the presence of NiONPs and MnO_2_NPs was studied in the presence of possible present excipients such as lactose, povidone, microcrystalline cellulose, magnesium stearate, anhydrous colloidal silica, red ferric oxide, and titanium dioxide. The tolerable values of the analytical drugs in the presence of interfering species were estimated. It was found that no significant interference was observed (Table 4). The outcomes indicated that the tolerable values of the foreign species gave a percentage error ≤5%. Accordingly, the suggested spectrophotometric method for the determination of the three selected drugs can be considered as the selective method.

### 3.5. Analytical Application

The developed spectrophotometric method was employed to detect LVX, CPX, and CTX in their bulk powder (Table 5). The obtained data were found to be 99.36 ± 0.9, 99.53 ± 0.6, and 98.94 ± 0.8%, for LVX, CPX, and CTX in the presence of NiONPs. Whereas, the percentage recoveries were found to be 99.70 ± 0.7%, 99.64 ± 0.6%, and 99.24 ± 0.8% for the three drugs in the presence of MnO_2_NPs, respectively. These outcomes revealed the high sensitivity of the developed method in the presence of metal oxide nanoparticles due to their large surface areas and high optical features.

The developed analytical method was applied to quantify the investigated drugs in their commercial dosage forms (Tavanic^®^ 500 mg LVX/tablet; Keflex^®^ 500 mg CPX/tablet; and Foxime^®^ 1 g CTX/vial), and the results were summarized in Table 6. The recorded results were found to be 99.64 ± 0.6%, 99.60 ± 0.4%, and 99.33 ± 0.7% for LVX, CPX, and CTX in the presence of NiONPs. Whereas, the percentage recoveries were found to be 99.48 ± 0.4%, 99.67 ± 0.6%, and 99.18 ± 0.8% for the three drugs in the presence of MnO_2_NPs, respectively. All results were statistically assessed using Student’s *t*-test and variance ratio F-test [68] and compared with those obtained from previously reported methods [69,70,71]. It was observed that the high sensitivity of the proposed spectrophotometric method for the determination of LVX, CPX, and CTX in the presence of NiONPs and MnO_2_NPs can be attributed to the catalytic properties of these nanoparticles. Metal oxide nanoparticles enhance the localized surface plasmon resonances and collective oscillations of conduction electrons that strongly couple to light at particular wavelengths and produce their extremely high optical properties. 

The high optical activity of these metal oxide nanoparticles was also due to their large surface area with tunable properties generating during their nanoscale synthesis and their ability to absorb the oxygen and sulfur groups of the analyzed drugs and enhance their spectrophotometric determination.

Furthermore, a comparative study was carried out between the results obtained from the suggested spectrophotometric method and the previously reported analytical techniques including spectrophotometric, electrochemical, and chromatographic methods. The outcomes have been summarized in Table 7. Practically, the suggested spectrophotometric methods using metal oxide nanoparticles displayed high sensitivity, cost benefits, and easy production, and the echo-friendly method depends on the preparation of nanoparticles using natural sources. Moreover, it is more accurate and precise and does not required higher technical skills when compared to other chromatographic or electrochemical techniques. 

## 4. Conclusions

The present study suggested a sensitive and selective spectrophotometric method for the detection of antibiotics (LVX, CPX, and CTX) in their pharmaceutical formulations. The detection is based on the enhancement effect of NiONPs and MnO_2_NPs on absorbance signals. The outcome of the investigation revealed that the increase in absorbance signals is proportional to the selected drug concentrations. The suggested method exhibits linear behavior over concentration ranges of 0.1–20, 1–80, and 0.001–100 µg mL^−1^ with least regression equations of Y = 0.0492C + 0.0334 (r = 0.9986), Y = 0.0319C + 0.0699 (r = 0.9999), and Y = 0.0304C + 0.0312 (r = 0.9995) for LVX, CPX, and CTX in the presence of NiONPs and Y = 0.0464C + 0.0956 (r = 0.9974), Y = 0.0163C + 0.0489 (r = 0.9995), and Y = 0.0321C + 0.0835 (r = 0.9998) for the above-mentioned drugs in the presence of MnO_2_NPs. The suggested spectrophotometric methods were successfully applied to determine the studied drugs in their pharmaceutical preparations. The recorded results were found to be 99.64 ± 0.6%, 99.60 ± 0.4%, and 99.33 ± 0.7%, for LVX, CPX, and CTX in the presence of NiONPs. Whereas, the percentage recoveries were found to be 99.48 ± 0.4%, 99.67 ± 0.6%, and 99.18 ± 0.8% for the three drugs in the presence of MnO_2_NPs, respectively. The high sensitivity of the proposed spectrophotometric method for the determination of LVX, CPX, and CTX in the presence of NiONPs and MnO_2_NPs can be attributed to the catalytic properties of these nanoparticles. Metal oxide nanoparticles enhance localized surface plasmon resonances and the collective oscillations of conduction electrons that strongly couple to light at particular wavelengths and produce their extremely high optical properties.

## Figures and Tables

**Table 1:** Optimized conditions for the determination of LVX, CPX, and CTX using the proposed spectrophotometric method in the presence of NiONPs and MnO_2_NPs, respectively.

Parameters	LVX	CPX	CTX
Wavelength (nm)	290	262	235
Volume of added nanoparticles			
NiONPs	0.2 mL	0.6 mL	0.4 mL
MnO_2_NPs	2.0 mL	1.0 mL	1.5 mL
Temperature °C	25	25	25
Response Time (min)	3	6	5

**Table 2:** Response data obtained from the determination of LVX, CPX, and CTX using the proposed spectrophotometric method in the presence of NiONPs and MnO_2_NPs, respectively.

Samples	λ_max_ (nm)	Beer’s Law Limit (µg mL^−1^)	Molar Absorbitivity (L. mol^−1^.cm^−1^)	Sandell’s Sensitivity (µg/cm^2^/0.001 Absorbance Unit)	Correlation Coefficient, (r)	Standard Deviation of Slope (S_b_)	Standard Deviation of Intercept (S_a_)	% SE *
LVX-NiONPs	290	0.1–20	17.9 × 10^3^	0.0203	0.9986	0.00074	0.00628	0.15
CPX-NiONPs	262	1.0–80	11.6 × 10^3^	0.0311	0.9999	0.00068	0.02809	0.05
CTX-NiONPs	235	0.001–100	14.5 × 10^3^	0.0033	0.9995	0.000311	0.013934	0.04
LVX-MnO_2_NPs	290	0.01–60	16.8 × 10^3^	0.0221	0.9974	0.00135	0.03609	0.08
CPX-MnO_2_NPs	262	0.1–160	5.9 × 10^3^	0.0610	0.9995	0.00016	0.01248	0.03
CTX-MnO_2_NPs	235	0.01–80	15.3 × 10^3^	0.031	0.9998	0.00025	0.009233	0.02

* SE represents the standard error.

**Table 3:** Validation data obtained from the determination of LVX, CPX, and CTX in bulk powder using the proposed spectrophotometric method in the presence of NiONPs and MnO_2_NPs, respectively.

Samples	Accuracy (*n* = 9)	Intra-Day (*n* = 3)	Inter-Day (*n* = 3)	Repeatability (RSD %, *n* = 6)	Robustness	Ruggedness
LVX-NiONPs	99.27 ± 1.4	1.3%, 1.7% and 1.5%	0.4%, 0.2% and 0.1%	0.6	99.38 ± 0.6	98.56 ± 0.8
CPX-NiONPs	99.59 ± 0.4	0.7%, 0.7% and 0.6%	1.5%, 1.6% and 1.3%	0.3	99.82 ± 0.7	98.52 ± 0.5
CTX- NiONPs	99.07 ± 0.8	0.5%, 0.6% and 0.6%	0.7%, 0.9% and 1.0%	1.1	98.89 ± 1.2	98.76 ± 0.7
LVX-MnO_2_NPs	99.77 ± 0.5	0.4%, 0.4% and 0.6%	1.1%, 1.2% and 1.4%	0.7	98.94 ± 0.8	99.23 ± 1.1
CPX-MnO_2_NPs	99.32 ± 0.7	0.9%, 0.8% and 0.7%	1.1%, 1.2% and 1.4%	0.3	99.45 ± 0.7	98.85 ± 0.6
CTX- MnO_2_NPs	99.17 ± 0.7	0.6%, 0.6% and 0.8%	0.9%,0.8% and 1.0%	0.8	99.85 ± 0.3	98.47 ± 0.6

**Table 4:** Effect of foreign species on the determination of LVX, CPX, and CTX using the spectrophotometric method in the presence of NiONPs and MnO_2_NPs.

Interference	LVX Tolerable Values		CPX Tolerable Values		CTX Tolerable Values	
	NiONPs	MnO_2_NPs	NiONPs	MnO_2_NPs	NiONPs	MnO_2_NPs
Lactose	100	200	80	100	100	140
Povidone	400	420	200	200	250	300
Microcrystalline cellulose	300	200	100	200	100	180
Magnesium stearate	600	600	400	300	300	340
Anhydrous colloidal silica	750	680	560	600	420	510
Red ferric oxide	800	700	600	580	350	200
Titanium dioxide	200	300	120	100	250	150

**Table 5:** Determination of LVX, CPX, and CTX in bulk powder using the proposed spectrophotometric method in the presence of NiONPs and MnO_2_NPs.

Samples	Taken Conc. Range (µg mL^−1^)	Found Range (µg mL^−1^)	% Recovery	Mean ± SD	*n*	Variance	%SE	% RSD
LVX-NiONPs	0.1–20	0.1–19.66	97.9–100.66	99.36 ± 0.9	7	0.81	0.34	0.91
CPX-NiONPs	1.0–80	1.0–79.25	99.0–100.5	99.53 ± 0.6	7	0.36	0.23	0.60
CTX- NiONPs	1.0–80	0.995–97.31	97.8–100.1	98.94 ± 0.8	7	0.64	0.30	0.81
LVX-MnO_2_NPs	0.1–60	0.099–59.85	99.0–100.5	99.70 ± 0.7	7	0.49	0.26	0.70
CPX-MnO_2_NPs	1.0–160	1.0–158.22	98.9–100.5	99.64 ± 0.6	7	0.36	0.23	0.60
CTX- MnO_2_NPs	1.0–80	0.991–97.02	98.0–100.3	99.24 ± 0.8	7	0.64	0.30	0.81

**Table 6:** Determination of LVX, CPX, and CTX in their commercial dosage forms using spectrophotometry in the presence of NiONPs and MnO_2_NPs, respectively.

Samples	Taken (µg mL^−1^)	Found (µg mL^−1^)	% Recovery	Mean ± SD	*n*	Variance	% SE	% RSD	Reference Method [69,70,71]	*t*-Test (2.228) *	*F*-Test (5.05) *
LVX-NiONPs	2.0–20	1.98–19.95	99.0–100.5	99.64 ± 0.6	6	0.32	0.23	0.57	99.43 ± 0.9	0.482	2.53
LVX-MnO_2_NPs	1.0–60	0.99–59.6	99.0–100.0	99.60 ± 0.4	6	0.16	0.16	0.40		0.682	4.00
CPX-NiONPs	5.0–80	5.0–79.8	98.0–100.0	99.33 ± 0.7	6	0.49	0.29	0.70	99.28 ± 0.6	0.341	1.31
CPX-MnO_2_NPs	10–160	9.98–158.5	98.8–100.0	99.48 ± 0.4	6	0.16	0.16	0.40		0.693	2.25
CTX-NiONP	5.0–80	4.96–79.34	99.2–100.6	99.67 ± 0.6	6	0.36	0.24	0.60	99.03 ± 0.7	1.724	1.00
CTX-MnO_2_NPs	5.0–80	5.02–79.21	97.8–100.4	99.18 ± 0.8	6	0.64	0.33	0.81		0.341	1.306

* Tabulated *t*- and *F* values at *p* < 0.05.

**Table 7:** A comparative study between the results obtained from the determination of LVX, CPX, and CTX using spectrophotometry in the presence of NiONPs and MnO_2_NPs, respectively and the previously reported analytical techniques.

Analytical Techniques	Reagent	Linear conc. Range µg mL^−1^	LOD	Reference
Spectrophotometry	LVX, Methanolic solution of perchloric acid	2.5–20	0.8 µg mL^−1^	[6]
	CPX, Oxidation using excess amount of N-bromosuccinimide	1.0–9.0	0.023 µg mL^−1^	[7]
	CTX, Metal Cu (II) complex	20–140	--	[8]
Fluorescence	LVX, Electron transfer mechanism quenched the fluorescence of GSH-CdTe QDs	0.73–7.30	1.53 ng mL^−1^	[9]
	CTX, Bromate-bromide and, acriflavine as fluorescent dye	0.1–3.0	0.013	[10]
Electrochemical	LVX, Nanocomposite of silver nanoparticles on a thin and porous nickel oxide film.	0.25–100 µM	--	[12]
	CPX, Disposable graphite electrode	0.5–4.0 mM	0.12 µM	[13]
	CTX, A multi-walled carbon nanotubes (MWNT) coated glassy carbon electrode	0.004–10.0 µM	1.0 nM	[14]
Chromatography	LVX, Acetonitrile and 0.01 M potassium dihydrogen aqueous solution (pH 3.4; 14:86 *v*/*v*)	0.1–12	0.05	[15]
	CPX, OPA: Acetonitrile (30:70 *v*/*v*)	100–500	0.357	[16]
	CTX, Methanol:Phosphate buffer (1000:130 *v*/*v*)	0.5–1.5	35.5 ng mL^−1^	[17]
Proposed method	Spectrophotometric measurement in the presence of NiONPs and MnO_2_NPs	0.1–20	0.07	LVX
		1.0–80	0.09	CPX
		0.001–100	0.0005	CTX

## Data Availability

All data of this study are included within the text.
